# Self-Confidence an Essential Element in the Character of the Ideal or Model Dentist

**Published:** 1881-04

**Authors:** Edgar Palmer

**Affiliations:** La Crosse, Wis.


### SELF-CONFIDENCE AN ESSENTIAL ELEMENT IN THE CHARACTER OF THE IDEAL OR MODEL DENTIST.

BY DR. EDGAR PALMER, LA CROSSE, WIS.

*Mr. President and Members of the Iowa State Dental Society:*

I can truly say that I feel highly honored by your invitation to contribute a paper and otherwise take part in the exercises of this, the eighteenth annual session of one of the most vigorous and talented societies in the country. It is indeed an honor which I desire to acknowledge in the most fitting terms of appreciation.

Nothing less than this sense of my obligation and the desire to make some show of an "offering" in return for the great benefits I shall receive from the essays and discussions of your learned men in this convention induces me to presume to attempt a literal compliance with your kind request.

I am indebted to your worthy corresponding secretary for having given me the cue to a subject for this occasion. In one of his excellent letters to me he makes use of the following language; an expression of sentiment, which, aside from its promptings to me, is too good to be lost. He writes: "We need to discuss, not more the practical methods of office work than the fundamental ideas of professional life, the basis on which the profession rests." The *ideal* dentist comes first into being, then the *real* and *actual* according to the pattern mentally evolved.

Men are, and become, what their aims and purposes make them. We need to get before the profession the true pattern of a dentist, a model to work to. This must be outlined in the mind of every aspiring dentist before he will rise to any high standard, and before he can help his professional brethren up higher."

Were I to attempt to give you my description of an "ideal" or "true" pattern of a dentist, I should expect him to possess as one of the important elements of his character, *self-confidence.* Not that blind, arrogant, assuming egotism which I am afraid adorns the surface of some in our profession; an egotism that sees no defects, weakness or failure in self, and no merits in others; but a quiet consciousness of inbred power; a calm, dignified composure of individual strength with the work to be performed.

Nothing worthy of note was ever accomplished by him who doubted his ability to perform the task he had undertaken. Great achievments are reached only by those who feel conscious of their own inherent power, and who "never falter or look back," when beset with difficulties, while any possibility remains that energy and diligence can remove them.

A weak, hesitating, wavering policy is an almost certain forerunner of defeat.

The great rulers of the world have been men who felt a power within themselves superior to courts, ministers and counselors; men who have had a reliance upon themselves rather than on deceitful and betraying advisers.

The dentist who best posesses this self-power is in the broadest sense a *ruler* in his professional life, and his responsibilities as such a ruler are greater than many of the thoughtless and indifferent have ever conceived.

His empire is not limited to his work room or office; it extends to the homes of his clients, and the power he has is felt wherever his subjects go. Neither is his responsibility limited to his ability for remedying existing evils; but he must so rule as to develop, not only a *passive* obedience in his subjects, but endeavor to rule them that they themselves may become rulers; controlling not only their own time and efforts in compliance with his commands, but the thoughts and actions of those at home, abroad, through childhood and youth, and all along the varying conditions of their lives, and so out upon an unborn generation, when he and his subjects will have ceased to exist.

In this age of apparent degeneracy of the human teeth, who shall say that these are not responsibilities which every conservative dentist should feel. Responsibilities which consciously or unconsciously test his allegiance to the true duties and functions of his office, and from which, if he be loyal to his subjects, he cannot shrink.

Who, then, among us, can question the dentist's need of this *consciousness of self-power* in his professional life?

Who has more need of strength of character and purity of motive?

He may possess enthusiasm, energy, industry, knowledge and mechanical skill; but if he lack this great confidence in his own ability to so use these faculties as to command confidence and insure obedience, he can hardly hope for complete success.

Admitting, then, the importance of this element, we may profitably inquire from whence it is derived, whence this all inspiring consciousness of self-power which furnishes the dentist abundant and never failing resources, and carries him through tempests of trials and discouragements? I answer, first of all,

SINCERITY.

Let his other qualifications be what they may, the dentist can not long hold in trust the faith and confidence of the people unless he brings to his work an evidence of good motives for engaging in his calling, and the most perfect honesty in executing his responsible duties.

Patients are critical observers and shrewd to detect evidences of deceit and hypocrisy, and if we have no higher interest in their welfare than that which directly affects our pockets, they will soon discover it and use it to our material disadvantage.

This is a quality which must not only be genuine, but *ever present* with the dentist and felt in the glance of his eye, the tone of his voice, in every word that he speaks, professionally, or in every act or operation he performs.

Closely allied to sincerity and partly dependent upon it is *the power of self control.*

Unlooked for perplexities, the innumerable vexations which daily occur, the stupidity of some and the malicious representations of others for whom we are called upon to work, we must be prepared to encounter without feelings of vexation, for so soon as the dentist loses his self control and pours out the bitterness of his soul, just so soon is he shorn of half his power; with a loss of temper his strongest line of fortifications has been swept away, and with it some of his self-respect and justly that of others.

To this quality we mention next

### A SOUND BODY.

An ancient writer says " A sound body is necessary to a sound mind."

It is a well recognized fact that our professional labors are not calculated to strengthen the frame or develop in us a healthy physical organism, without which the dentist is by no means in proper condition for professional duty, nor can he expect to rise superior to his physical environments in his intellectual endowments; hence it is true that our profession as well as students of any other science should seek to enlarge the sphere of intellect through a proper training of the muscles and the development of physical power.

I believe it to be the neglect of hygienic laws and the disregard of these important matters of *pure air*, *recreation*, and proper exercise of the unused muscles of our bodies, that causes so many of our professional men to break down under what others, who are more observant of the claims of nature, perform with ease and comfort.

And in proportion as the dentist's mental powers and capabilities are quickened and strengthened by a strong and healthy body, just to such a degree will his energy, ambition and self-confidence sink when these physical forces are withdrawn.

### THOROUGH SCHOLARSHIP

is no longer considered indispensable to the ideal or model dentist. Dentistry, with her numerous incorporated schools and a literature rapidly increasing in volume, interest and importance, is making good progress towards rescuing it from the grasp of ignorance and empiricism, and developing agencies for achieving scientific methods and practical results, by which the health, happiness and longevity of the human race is promoted, and by virtue of its functions and its attainments, is taking a place as "a peer among the arts beneficent" — arts whose aim is to relieve human suffering and promote the welfare of the human family.

Text books, however, contain but the skeleton of science and too often (as I believe to be the case in our own profession) a very imperfect one at that. There is a broad ground to be explored outside of the text books before the dentist should don the full professional garb. Says Prof. Coy: "Only time, study and hard rubs can sift and utilize the wild theories and overweening confidence natural to the novice or newly-fledged enthusiast, and make him the real possessor of that wisdom and foresight which the wants of the age and the needs of humanity in this specialty requires."

Our vocation is being recognized as a branch of the healing art, requiring not less in preparation or scholarship than other learned professions, and is no longer considered dispensable or of small importance; and those who adopt it should do so with right motives and be stimulated by that pride of their calling which seeks not only to guard its good name among the arts and sciences, but to elevate its standard by a broadening of its usefulness in the interests of a generous humanity; and he who is thus imbued with a just conception of the degree of culture, integrity and humanity demanded, and realizes that his mind, conscience and soul are made powerful through discipline, polished by contact, and attuned in harmony with the noblest aspirations of man's nature, is at once the possessor of that mental power and self-confidence which defies the vulgar shams of ignorance and pretence, and carries him above and beyond the narrow, selfish, mercenary practices, which would disgrace his calling and destroy his power and influence.

Another element, and one of the first laws of success with men of every calling at the present day, when so many matters are clamoring for attention, is

CONCENTRATION.

It has been justly said that "one talent well cultivated, deepened and enlarged, is worth a hundred shallow faculties."

He who singles out dentistry as a specialty must pour into it the whole stream of his activity. All the energies of his hand, eye, tongue, heart and brain — to bend all his energies to one point, looking neither to the right nor to the left.

"The day of universal scholars is past." The range of human knowledge has become of such magnitude that no brain can grapple with it; and however attractive or inviting is the contemplation of beautiful things outside of professional duty, the wisdom of the dentist is usually proven and his practical proficiency best shown by thus bending all his energies in the one direction of his specialty, and endeavoring to make those energies develop higher capabilities, which become — when thus strained or under such pressure, as under conditions of necessity — a wonderful educator, a wonderful enlarger and quickener of the faculties, and likewise the most solid ground-work for self-confidence and mental power.

Possessing the elements which enables him to have a well grounded confidence of success, the dentist ought to acquire a love for his duties, enjoy his labors in the interests of his science, realizing that they are not humble and belittleing, but *grand* and *humane*.

No sculptor ever touched chisel upon such noble and profitable material. No musician ever touched the keys of an instrument more complicated and delicate.

No science of human government has the statesman ever explored so intricate as that which teaches the true principles and methods by which he is governed in his dealings with the manifold forms of complicated cases presented to him, and when the professional life of the dentist reflects the image of our "ideal" or "model" through the possession of such elements, one of which I have briefly mentioned, we can feel assured that his work, like the sculptor's chisel upon the marble pile, will bear his faithful impression long after his labors have ceased, and the influences of his self-confidence and mental power will be felt even after these types of material permanence have melted away.

